# Analysis of selected policies towards universal health coverage in Uganda: the policy implementation barometer protocol

**DOI:** 10.1186/s13690-018-0258-4

**Published:** 2018-02-15

**Authors:** Charles Hongoro, Elizeus Rutebemberwa, Thembinkosi Twalo, Chikondi Mwendera, Mbuyiselo Douglas, Moses Mukuru, Simon Kasasa, Freddie Ssengooba

**Affiliations:** 1 Population Health, Health Systems and Innovation, Human Sciences Research Council (HSRC), 134 Pretorius Street, Private Bag X41, Pretoria, 0001 South Africa; 2 School of Health Systems and Public Health, University of Pretoria, Pretoria, South Africa; 3 Makerere University School of Public Health, Kampala, Uganda

**Keywords:** Policy implementation barometer, Balanced score card, Uganda

## Abstract

**Background:**

Policy implementation remains an under researched area in most low and middle income countries and it is not surprising that several policies are implemented without a systematic follow up of why and how they are working or failing. This study is part of a larger project called Supporting Policy Engagement for Evidence-based Decisions (SPEED) for Universal Health Coverage in Uganda. It seeks to support policymakers monitor the implementation of vital programmes for the realisation of policy goals for Universal Health Coverage. A Policy Implementation Barometer (PIB) is proposed as a mechanism to provide feedback to the decision makers about the implementation of a selected set of policy programmes at various implementation levels (macro, meso and micro level). The main objective is to establish the extent of implementation of malaria, family planning and emergency obstetric care policies in Uganda and use these results to support stakeholder engagements for corrective action. This is the first PIB survey of the three planned surveys and its specific objectives include: assessment of the perceived appropriateness of implementation programmes to the identified policy problems; determination of enablers and constraints to implementation of the policies; comparison of on-line and face-to-face administration of the PIB questionnaire among target respondents; and documentation of stakeholder responses to PIB findings with regard to corrective actions for implementation.

**Methods/Design:**

The PIB will be a descriptive and analytical study employing mixed methods in which both quantitative and qualitative data will be systematically collected and analysed. The first wave will focus on 10 districts and primary data will be collected through interviews. The study seeks to interview 570 respondents of which 120 will be selected at national level with 40 based on each of the three policy domains, 200 from 10 randomly selected districts, and 250 from 50 facilities. Half of the respondents at each level will be randomly assigned to either face-to-face or on-line interviews. An integrated questionnaire for these interviews will collect both quantitative data through Likert scale-type questions, and qualitative data through open-ended questions. And finally focused dialogues will be conducted with selected stakeholders for feedback on the PIB findings. Secondary data will be collected using data extraction tools for performance statistics.

**Discussion:**

It is anticipated that the PIB findings and more importantly, the focused dialogues with relevant stakeholders, that will be convened to discuss the findings and establish corrective actions, will enhance uptake of results and effective health policy implementation towards universal health coverage in Uganda.

## Background

Policy implementation remains an under researched area in most countries and it is not surprising that several policies are implemented without a systematic follow up of why and how they are working or failing [1, 2]. It is for this reason that a project entitled “Supporting Policy Engagement for Evidence-based Decisions (SPEED) for Universal Health Coverage (UHC) in Uganda” was launched by the School of Public Health at Makerere University (MakSPH) in 2015. The overall objective of SPEED Project is to strengthen capacity for policy analysis, implementation monitoring and analysis of impact and thereby contribute to accelerating progress towards UHC in Uganda. This paper describes the proposed Policy Implementation Barometer (PIB) survey which is a mechanism to reveal gaps in policy implementation and thereby provide feedback to the decision makers about the implementation of a selected set of policy programmes for UHC. The feedback mechanism will include policy engagements to foster corrective action in areas where such is required. The SPEED project seeks to conduct 3 barometer surveys over its 5 year life span (2015–2020). Lessons from the first wave will advise the implementation of the policies, hence a second barometer survey using the same tools and approach will be conducted in the third years to evaluate progress and change in the implementation of the policies. Finally, the last barometer wave will be conducted in the fifth year, which is the final year of the project. By using the same tools and methods, this evaluation will detect the overall outcomes of the project.

## Purpose

Uganda has developed several acclaimed health policies to transform the wellbeing of its population. However, the intended results have not always been achieved due to inadequate implementation [3]. The identification of policy implementation failures and suboptimal performance through monitoring and accountability for policy implementation is mostly downward looking – mostly identifying frontline failings at service delivery levels. There is limited research and tools to monitor upstream actions (e.g. financing, partnerships and support systems) that are vital to the performance of frontline functions. This partly arises from the result-orientation in research funding and methodologies that are well established for the downstream outcomes and less well tuned for upstream and mid-level actions. Upstream monitoring is predominantly relational and process-oriented with fewer research tools to make meaning of the complex relationships that underlie up-stream functions. The health system research agenda has brought to the fore processes that support service delivery [4]. Once a policy is passed, there are often weak mechanisms to provide systematic feedback to the decision makers about implementation progress [1, 2]. Where it exists, it is often about what frontline actors should do to improve policy outcomes but less focused on the upstream actions that support implementation processes that may constrain implementation such as organizational architecture and partnerships, workforce capabilities, costs and financing, mobilization and compliance of policy beneficiaries. There are few tools that allow upstream policy makers and policy advocates to monitor implementation and to mobilize appropriate and continued policy support during the implementation phase.

There is limited research that tracks the implementation phase of policies for the benefit of informing corrective actions [1, 2]. This gap manifests in delayed corrective actions for policy implementation processes or gradual abandonment of policies that would otherwise transform the health and welfare of communities. Although some programme monitoring tools exist, these mostly serve a technocratic objective, mostly organised on the basis of project silos and internal indicators of project management. Evidence emerging from HIV programmes show that active implementation tracking of project indicators has a tendency to divert the health systems capabilities towards a couple of projects to the neglect of other vital policy programmes [5].

The specific objectives of the PIB study are (1) To assess the perceived appropriateness of policy programmes implemented to address identified policy problems (e.g. policy goals, strategies, standards and processes of decision making); (2) To assess, using priority parameters, the extent to which the prerequisites for implementation are established (e.g. awareness of policy objectives, resources provision, network of role bearers, community demand, policy champions); (3) To determine the enablers and constraints to implementation of the selected policies (e.g. workforce, finance, and medicines and the interdependencies of action network); (4) To compare on-line and face-to-face administration of the PIB questionnaire among target respondents (ease of access, response rate, costs); and (5) To document stakeholder responses to PIB findings with regards to policy implementation, awareness and actions. This will include dialogue on content such as implications and recommendation, actionable findings and role accountability collaborative actions.

## Conceptual frameworks

Policy implementation is a complex process. First, it requires a good understanding of what a policy is, and secondly the various approaches to translating policy into programmes and activities towards the expected results. The succeeding section unpacks these issues and draws some insights towards the development of this theory-driven protocol for conducting a PIB in Uganda which may be applicable to other low and middle income countries.

## What is policy?

Policy is variously defined to mean “statement of intent” (e.g. to eradicate ebola by year 2020). The definition of policy also needs to be understood in terms of its context and perspective, for example, whether it is at government or organisation level. At governmental level, a policy could be defined as the declared objectives that the government seeks to achieve for the benefit of its citizenry. The commonly used definition by health policy scholars is that policy is a product of ‘the interplay between institutions, interests and ideas’ [6]. For this study, health policy is broadly defined to include a plan, principle or guiding decisions that provide a framework for actions.

There are different levels of policy, often referred to as: macro that relates to governmental or systemic level with a broader or national reach; meso that relates to organizational or programmatic levels (e.g. at district or hospital level); and micro that relates to the clinical or professional practice level guidelines and controls [7]. We seek to explore specific issues at these levels particularly as they relate to appropriateness of policy design and content, and how specific policies are translated into action to achieve desired results at district and facility levels – hence policy implementation. This obviously assumes a top-down policy development and implementation approach.

## What is policy implementation?

**Fig. 1:**
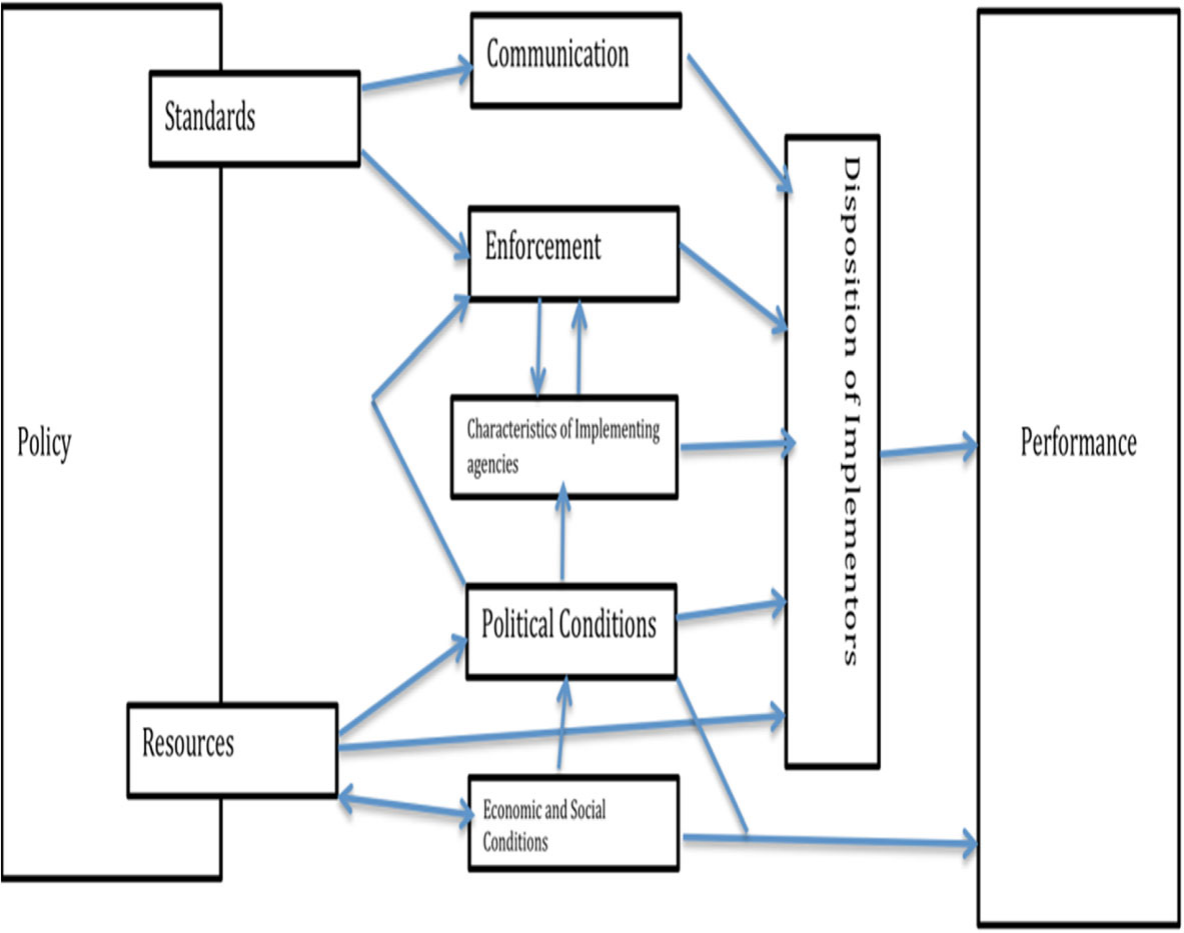
The 8 factors influencing of Policy Implementation (Source: [12])

Policy implementation is described as what happens between policy expectations and policy results [8–10]. What happens between ‘what is expected’ from a policy and the outcomes of the policy includes actions and inaction by public and private individuals that lead (or not) to the realisation of the policy objectives [11, 12]. Research in this field is driven by a need to understand and explain the variables that affect the translation of policy as intent to policy as practice [13]. Mazmanian and Sabatier [12] proposed a conceptual model (Fig. 1) in the analysis of the variables that shape the relationship between policy as intent and policy as practice, and these have guided the general approach in this study protocol and are described below:

### Policy resources

Policies require resources, such as financial and human, for the administration, support, and enforcement of the implementation process. The availability and nature of policy resources influences the predisposition of policy implementers, who are responsible for executing the policy objectives [13]. If resources are inadequate policies are likely to be inadequately implemented [14]. Weaver [14] argues that oftentimes policy makers do not factor in resource needs and how to acquire them in formulating policies and this is often left to the bureaucrats as implementers to deal with, which can substantially slow down the implementation process or derail it at worst.

### Policy standards

Policy standards specify the expected policy goals and what is expected of each actor within the policy implementation process. They also determine requisite tools for the enforcement to ensure compliance with policy objectives and therefore, may take the form of legislation, regulations, technical guidelines, and standard operational procedures. They also describe how the policy will be enforced. To aid the implementation process, these need to be adequately and timeously communicated, understood, and accepted by the implementers. The quality, clarity, consistency and accuracy of these standards determine the extent to which the policy is effectively articulated [11].

### Communication

This is important in ensuring that the policy standards are clearly understood by the implementers. The policy standards should be articulated in such a way that it is clear to the implementers what is expected of them, leaving no room for ambiguity. Where ambiguity is left unaddressed it opens up the policy implementation process to different interpretations by the implementers who assume the role of street level bureaucrats leading to uneven policy outcomes within a country or at worst objectives contrary to the policy being realised [15]. Problems may also arise when the government representatives at the meso-level bring different interpretations of the policy to the implementers or bring their own judgment to bear on the policy implementation process by emphasizing different aspects of the policy leading to gaps in the policy implementation process at the grassroots level [11].

### Enforcement

Mechanisms and procedures for monitoring the enforcement of the policy should be in place with deviations promptly attended to. Policy enforcement may be normative, remunerative or coercive depending on the context. The level of enforcement will be determined by factors such as power relations amongst the different policy actors, access to political resources, ability to monitor progress of the policy implementation process as defined by the availability of technical staff and financial resources to do so, reporting structures and mechanisms [16, 17].

### The characteristics of the implementing agencies

These relate to the nature of the organisations and their relationships or interdependences. Whether private or public, the organisational network for policy outcomes may work through collaboration or competition. It could also include the level and type of resources available to the organisation network for the implementation of the policy [18].

### Disposition of implementers

The implementers assume the role of street level bureaucrats that determine the fidelity of the policy implementation process. There are many factors that influence the disposition of implementers such as their beliefs and attitudes towards the policy, their level of training, their level of cognition of what the policy requires, and whether or not there are policy champions in their midst. For example, the implementation of termination of pregnancy policy in South Africa was initially hindered by implementer beliefs and attitudes that affected its actual availability in some areas [19].

### Political environment

The level of political support for policies will influence in a significant way whether and how the policy is implemented. This was seen in the HIV response during the early 2000 in South Africa and in Uganda since 1986 [20, 21]. In South Africa, the Mbeki administration’s was criticized for its policies that denied people access to anti-retroviral drugs (ARVs) which exacerbated the HIV/AIDS death rate. Conversely, extensive support and political will to combat HIV in Uganda is often cited as the main reason for the dramatic reduction of HIV prevalence between 1990 and 2008. The political environment creates a conducive space, avails policy champions, and makes resources and community mobilization feasible.

### Economic and social conditions

The level of economic development and the fiscal space within a country will determine the level of resources that are available to implement any policy. Fiscal space is the budgetary room for a country or government to allocate funds from the national fiscus in a sustainable way [22].

## Assessing policy implementation

Good policies are usually developed at central level with the assumption of being automatically translated to results by implementers, which creates a disjoint that leads to an implementation gap [23]. Challenges to the implementation of health policies have been acknowledged in various settings and require evaluation to identify them [24, 25]. Assessment of these challenges requires a systematic approach for early recognition and the application of remedial measures. Similar studies as proposed in this PIB have been conducted by the USAID Health Policy Initiative in the implementation of HIV & AIDS – related policies in China, Indonesia, and Vietnam [26]. The initiative developed a systematic approach for assessing and reducing the barriers called the Policy Implementation Barrier Assessment (PIBA), which focused on concepts of motivation, power, information, interaction, and networks as critical elements. Data was collected by qualitative methods through focus group discussions and in-depth interviews with purposively identified policy makers and programme implementers. Finally, an iterative feedback session with stakeholders was conducted to brainstorm and identify remedial strategies. The assessment identified both upstream and downstream factors affecting policy implementation. This approach thus, provides a framework for assessing barriers to policy implementation and the consideration of contextual factors in this process.

This PIB research draws from political science practices where voter barometers have been used to gauge campaign messages, popularity of policy options and satisfaction of political reforms. It also draws heavily from the business and policy implementation research literature which emphasises the importance of understanding the theory of change (TOC) of a policy, determinants of change, implementation objectives, networks of implementers, targets, contexts, strategies for implementation and implementation impact.

Kaplan and Norton [27] described “a multidimensional framework for describing, implementing and managing strategy at all levels of an enterprise by linking objectives, initiatives, and measures to an organisational strategy,” which encompasses both financial and non-financial parameters. For instance, in Canada key parameters used for the balanced score card aimed at quality improvement included 1) resource and services, 2) community engagement, 3) integration and responsiveness and 4) health determinants [28]. The barometer-like assessments, sometimes branded as “Balanced Score Card” or “report card” have been mostly applied in developed countries [28–30] and the private sectors. More recently, similar assessments have been successfully applied in low and middle income countries to assess health service performance [31–33]. The PIB measurements are informed by two analytical frameworks: Firstly, on the score card approach which tends to focus on a few but critical dimensions of programme implementation [28, 32, 33]; and secondly, on the policy ingredients approach or the health system approach [33, 34] in which selected policies are unpacked into their essential strategies and activity sets [35, 36].

**Fig. 2:**
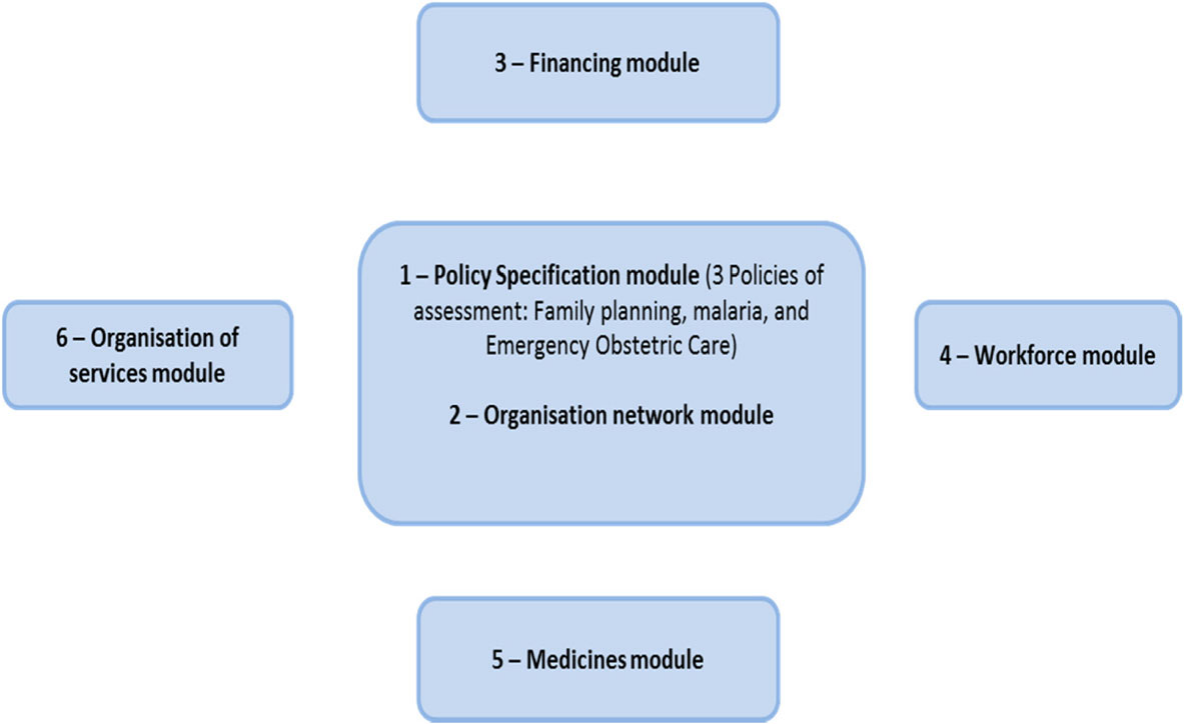
The six priority parameters of the Policy Implementation Barometer

For our purpose, there are six priority parameters to be assessed as illustrated in Fig. 2. These have been synthesised from the parameters discussed above and organised along the major strategic clusters in the practice management programmes. An additional coverage module relating to universal health coverage is added.

Unlike detailed explanatory studies, barometer-like assessments aim to provide quick diagnostics to support decision makers with general pointers of parameters that need detailed information or actions. The main advantages are worth highlighting below:

First, such assessments are a management tool that allow for simultaneous consideration of various policy or performance domains. It is therefore, possible to assess implementation of multiple policy interventions. Second, when there is stakeholders’ buy-in of the assessment framework, it is easy to implement since data access and stakeholder participation become easier. However, the barometer assessment framework does not work well where the policy under consideration does not have a clear theory of change. This is vital in understanding and explaining the observed performance. For this study, policy implementation tools such as strategic plans, budget framework papers, programmes or projects will all be used in the document review to clarify the implementation tools.

The specific focus areas for each parameter will be assessed at health system levels. The contents of the assessment framework need to be negotiated and agreed to by stakeholders in order to have legitimacy. Weir et al. [28] suggest a set of interrelated questions that should guide the assessment framework, and these include the following (Table 1):

**Table 1:** Barometer Questions

1. Is the policy appropriate for the identified problem?
2. What is the policy design and theory of change?
3. Is the policy being implemented as designed?
4. What is the capacity and readiness for policy implementers at national, district and facility levels?
5. How are vital resources – funds and other systems resources mobilized for implementation?
6. How adequate are the external dependencies for duty bearer agencies?
7. What is the perceived or verified level /degree/extent of implementation among duty bearers?
8. To what extent are the expected policy benefits being generated?
9. What is the perceived or verified extent of engagement of beneficiaries or policy advocates in the implementation arrangements for voice, effectiveness and responsiveness?
10. What are the enablers and constraints to policy implementation?

## Study objectives

### Main objective

Through the PIB, the study aims at assessing the implementation of selected health policies for Universal Health Coverage (UHC) in Uganda.

### Specific objectives include:

1. To assess the perceived appropriateness of implementation programmes to the identified policy problem.
2. To assess, using priority parameters, the perceived extent of implementation for selected policies.
3. To determine the enablers and constraints to implementation of the selected policy.
4. To compare on-line and face-to-face administration of the PIB questionnaire among target respondents.
5. To document stakeholder responses to PIB findings with regard to corrective actions for implementation.

## Methods

### S**t**udy setting

The policy implementation barometer will focus on three levels of policy implementation namely national (macro), district (meso) and facility (micro) levels. The fundamental assumption is that most policies in Uganda require supportive actions at these three levels. This frames the policy setting and hence the expected implementation variables of interest for the barometer.

### Criteria for choosing policies to assess

There are many health policies in Uganda but this barometer will focus on selected priority policies for assessment in different phases or waves (See Table 2). The first wave of the barometer will focus on policies related to family planning, emergence obstetric care and malaria policies. Policies to assess were selected based on the criteria described below. In subsequent barometers (in year 3 and 5) policies to assess will also depend on consultation with stakeholders which include the Ministry of Health and government using the same criteria as well as the experience of the first round assessment.

**Table 2:** Potential Polices for the Barometer

To ensure coverage of the whole health value chain, policies will be selected on the basis of their position in the value chain: (A) Prevention, (B) Treatment, (C) Follow up.
1) HIV policies
- Prevention of HIV e.g. ABC, PMTCT, etc.
- Treatment of HIV patients e.g. ART policies over time
- Follow up policies e.g. management of chronic illnesses, financing, management
2) Malaria policies
- Prevention of malaria policies e.g. insecticide-treated nets (ITNs) and Insecticide Residual Spraying (IRS)
- Treatment of malaria policies e.g. Antimalarial drug policies
- Follow up policies e.g. financing, management
3) Maternal mortality policies
- Prevention of maternal mortality e.g. maternal mortality reviews
- Treatment of pregnant patients e.g. Emergency obstetric care
- Follow up policies e.g. Policy on skilled birth attendance, financing, management
4) Family Planning
- Prevention of unplanned pregnancies e.g. contraceptive security
- Treatment of involuntary pregnant patients e.g. counselling, reproductive rights awareness
- Follow up policies e.g. service delivery and access, financing, management and stewardship
5) Child Health
- Prevention of child illness e.g. immunisation
- Treatment of child patients e.g. facilities, EPI
-Follow up policies e.g. Integrated Community Case Management (ICCM), financing, management

### Criteria for selection of policy for assessment

1. It is a topical policy under implementation in the country,
2. The policy has a specific policy history or age (based on when it started),
3. The policy is a priority to SPEED project target group in terms of need for evidence to support implementation decisions and or scaling up of activities,
4. The policy is clearly articulated and / or written, and
5. The policy is multidimensional to allow for multiple stakeholder engagement around a set of related themes rather than a narrow or vertical programming with less integration in the general health system.

### Study design

There will be three PIB waves. The first wave is the initial situation analysis survey which will assist the implementation of policies in the project’s lifespan. The second wave will be conducted in the third year of the project and the last wave will be final evaluation. Data collection will utilise the same tools and approaches in order to capture any changes hence, this PIB will be a longitudinal survey where both qualitative and quantitative data collection methods will be employed. The PIB study will be descriptive and analytical by focusing on selected policy cases with multiple assessment domains as outlined in Fig. 2.

### Data and data collection methods

The study will collect both primary and secondary data.

#### Primary data

Both qualitative and qualitative data will be collected through a structured questionnaire with open and close ended questions administered to relevant stakeholders at macro, meso and micro-level of policy implementation (Table 3). The face-to-face collection of primary data will be facilitated by research assistants who will be trained on how to administer the questionnaires. Five research assistants will be deployed in each district. After completion at facility and district level, a selected number of research assistants from this pool will be assigned to conduct the interviews at national level.

**Table 3:** Sample size estimates of key informants at various targeted levels

Data modules	Data source (10 districts) for the first round.	Overall sample size	Country specific sample sizes			
			National	District	Facility	Documents & HMIS-2 data
	*Study units*		*3 policy Areas*	*10 districts*	*50 facilities*	
	*Participants*					
Policy specific module 1. Malaria 2. Emergency obstetric care 3. Family planning	MOH programme officials National programmes DHMT and CSOs In-charge of facilities Programme managers in projects Health workers with related policy experience/roles	170	120 (40 per policy domain)	200 (20 per district	250 (at least 3 persons per facility)	• Programme documents • Policy documents • HMIS trends in the last 2 yrs. • Evaluations documents • Published literature • Government reports • Likert scale interviews with some open-ended questions • 6 modules of questions
Total unique respondents		570	120	200	250	
*Face-to-face interviews*		235	60	100	125	
*On-line interviews*		235	60	100	125	

##### Quantitative data

The likert scale-type options will be used for key measures of implementation status. The three questionnaires covering the three policy domains of family planning, malaria and emergence obstetric care (annexes 1, 2 and 3) are structured to include issues of policy resources, policy standards, implementation processes and other contextual factors that mediate policy implementation, which are further summarised in Table 4 as a framework for organising the assessment of the policy domains. Each questionnaire has six modules and addresses objectives 1 to 3. The first module is about policy specific details i.e. the policy objectives, and related programmes and plans that guide the implementation. The programme activities were used to generate six generic modules for assessment in Fig. 2. In the first module, all the selected policies will be assessed on the basis of their unique policy aspects.

**Table 4:** Framework for organizing the assessment domains for the- first wave of Policy Implementation Barometer

All Policy domains	Level of analysis	Policy resources (inputs)	Policy standards (process)	PIB indicator categories	Data sources
1. Family Planning 2. Malaria 3. Emergence obstetric care	Macro level	Availability of: • financial resources; • human resources (admin and technical staff); • other resources (equipment, supplies, medicines, etc.); • implementing agencies	Availability of: • regulations, • technical guidelines, • Standard Operating Procedures (SOPs); • Communication plan; • Enforcement mechanisms (normative, remunerative or coercive); • Policy champion; • Political resources (oversight committees, etc.); • Reporting structures and mechanisms	• Resources and services indicators • Community engagement indicators • Integration and responsiveness indicators • Family planning indicators • Malaria program coverage indicator *Emergency obstetric care* • Emergency obstetric care indicators • Policy champion; • Political resources (oversight committees, etc.); • Reporting structures and mechanisms	• Documents review • Key informant interviews • Interviews with implementers (all levels) • Secondary data (studies, surveys, routine health information, etc.
	Meso level	Same as above	Same as above	Same as above	Interviews Routine data
	Micro level	Facility level data - similar to the above	Facility level guidelines, SOPs, etc.	Facility level	Interviews Routine data

##### Qualitative data

As quantitative findings provide the status levels of the study variables, qualitative findings seek to provide the explanations/stories behind these levels. Qualitative data will be captured through the open-ended questions that will explore the explanations for the implementation status of a particular policy and where necessary solicit corrective strategies from the participants. The purpose of qualitative research approach is to provide a chance to the participants to share their lived experiences which may not be captured through quantitative methods. They provide an insight of contextual factors that affect the implementation of specific activities. Hence, the assessment of implementation processes will include the perspectives and experiences of strategically positioned practitioners and managers of these routine processes and arrangements such as financing, workforce, medicines and service delivery. A general question to solicit the ideas will be posed in form of ‘what are the main issues factors and actions needed to improve the shortcomings in the issues assessed?’

### Secondary data

Collection of secondary data will be conducted by the authors of this protocol. Each author will be tasked to review specific documents based on their research background.

Collection of secondary data will largely be through extraction from routine health information system (DHIS-2) at Ministry of Health and existing survey data in the three policy study areas. This analysis will also be done to include policy-wide or programme-wide information from evaluation reports, routine information systems and survey reports or recent publications as applicable. For example, the prevention of malaria programmes will include coverage and effective use of insecticide-treated nets (ITNs) and Indoor Residual Spraying (IRS). For malaria policy indicators will include things like antimalarial drug availability at frontline facilities. Emergency obstetric care and family planning policies will include the dimensions indicated in Fig. 2.

#### Sampling procedure

**Fig. 3:**
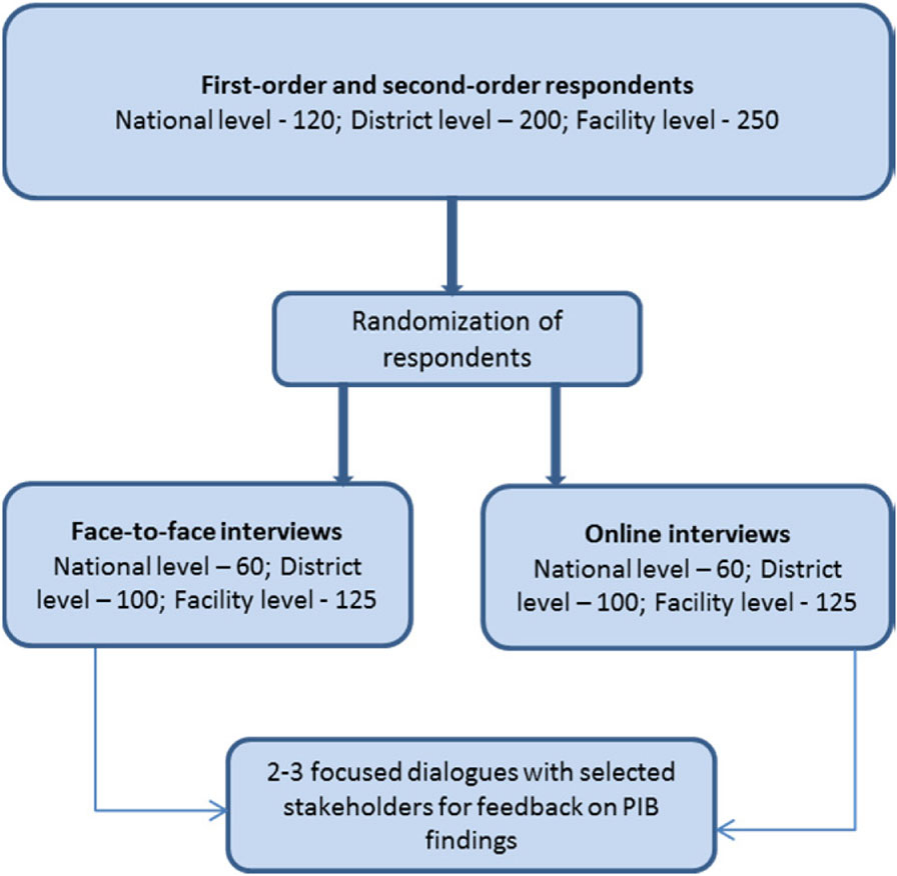
The selection flow of respondents

As highlighted in Table 3, it is anticipated that a total of 570 respondents will be interviewed at national, district, and facility levels. The sampling approach for these respondents will be based on two orders. Figure 3 illustrates the sampling stages and the randomization in the questionnaire administration.

##### First-order respondents

This includes respondents that are involved in or close implementation of the policy activities and hence they will be purposefully identified at the three levels. For example, at the national level, the first-order respondents will be those with formal mandate for implementing the selected policies – i.e. programme officials and national committees for 1) Malaria Control Programme and 2) Maternal and Child Health and Family Planning, and district level respondents will comprise the district health management teams (DHMT) and from the selected health facilities.

##### Second-order respondents

For each selected policy, a stakeholder mapping will be embedded in the interview (module 2) to identify the key stakeholders at national, district and facility level that are involved in or closer to the implementation of the selected policies. The respondents from this order will be identified by the first-order respondents. The most critical consideration in the selection of second-order respondents is that they must be engaged in an essential way in the implementation of the selected policy or programmes activities and be listed at least twice among the first-order respondents. Organisations mentioned/listed more than twice will form the sampling frame for the second-order interviews and 20 of these will be approached for barometer interviews.

Module 2 also collects information about the main roles played by these stakeholders.

Overall the anticipated sampling frame will include: MOH programme officials; National programme officials; district managers including (district Health Management Team (DHMT) and managers of district programmes that support the selected policy areas (malaria emergency obstetric care and family planning); Non-DHMT respondents will be identified by way of a rapid district level mapping of organisations that support these policy areas and this will be conducted with the guidance of the district health office (DHO) (module 2 on organisational networks). For each district, interviews will be conducted at one hospital (referral or general hospital), one Level IV health center and two Level III health centers, and two Level II health centers. This sample frame and procedures will reflect both the public and private facilities in the selected district. Where the numbers of facilities exceed those required for the sample, we will do stratified sampling within each level of facilities.

For objective 5, we plan to learn from the process of dissemination of the PIB findings. The main methods of data collection will involve the active documentation of stakeholder reactions and responses to the findings. The questions they ask about the finding and the ideas they propose about PIB and about corrective actions to improve implementation of the policy programmes. We aim to organise 2–3 dissemination meetings, and 2–3 focused dialogues with sub-groups of stakeholders on key issues that emerge from the PIB. We will use a structured narrative to capture the main aspects of responses to the PIB findings. In cases in which dissemination happens using mass media (radio, TV), we will also note the comments and questions raised and or the proposed ideas from the media/public. These will be analysed and used to improve the PIB tool and to produce an “ideas brief” that will complement the barometer findings. The brief will be shared with the target group and also help in interpreting findings or making plausible recommendations.

#### Questionnaire administration – On-line and face-to-face interviews

Through the two identified approaches, we seek to compare the on-line and face-to-face administration of the PIB questionnaires among the target groups. The purpose is to find the most effective way to interview respondents at the national, district and facility levels for the PIB. To inform future barometer surveys and to generate panel data to track trends in the key implementation domains, we will compare response rates, interview completeness and costs between on-line and face-to-face. A pool of respondents and their telephone contact at each level (national, district and facility) will be generated. From Table 3 half of the respondents will be assigned to either face-to-face or on-line interviews. Those that are assigned to fill on-line questionnaires will be requested to provide email addresses so that they can be invited to participate in the study. Within the email, a web-link to the survey will be provided. The survey will be hosted on the MakSPH server. To compensate respondents for the data and connectivity costs of filling the survey, a token of appreciation will be provided.

##### At national level

respondents will be randomised by policy cluster (e.g. malaria, Family planning and emergency obstetric care). As we target to interview 120 persons at the national level i.e. 40 agencies/departments in each policy area, random allocation will be done to assign half to face-to-face interviews and half to on-line interviews. Before the random allocation, we will organise the second order organisations according to three policy domains to ensure a more balanced response across the three policy areas.

##### At district level

Overall 10 districts will be selected from district clusters (strata) ranked in accordance with the district league table and will be shared with Ministry of Health during the inception stage of the survey. Following this general framework, the selection criteria below was generated and followed in the selection of the districts:

i. Top fifteen performing districts according to the following clusters: national, regional, hard- to-reach districts, new districts and districts without regional referral hospitals,
ii. Bottom fifteen performing districts in the same clusters in criteria (i) above,
iii. Old districts expected to have fairly resilient health systems,
iv. Peri – urban versus rural districts, and
v. Districts where MakSPH has previously or is currently conducting activities with potential synergies with the PIB.

Using the above criteria, the districts selected to participate in this survey are: Arua, Jinja, Tororo, Hoima, Gulu, Kabarole, Kibuku, Kitgum, Ibanda and Wakiso.

Since there are 10 targeted districts for the PIB, respondents will be randomized using the district as a cluster – 5 districts for online and 5 district for face-to-face interviews. In addition to administration of the questionnaire at the district level, relevant routine data that cannot be collected at the national level will be also be collected.

The face-to-face survey will be conducted in 5 districts to be randomly selected from the 10 selected districts. The remaining districts will be assigned to on-line survey. In each district, interviews will target about 20 district level managers, and 20–25 facility level managers. In total (Table 3), we expect a sample of 570 respondents at the different levels of implementation.

### Data analysis and displays

All quantitative and qualitative data collected from secondary and primary sources will be used to construct the barometer measurement metrics for the selected policies or policy domains. Descriptive and comparative statistics will be calculated to show implementation progress by policy/programmeme areas. The analysis will be simple enough to be understood by decision makers but clear enough to show actual implementation gaps. Qualitative data collected through open-ended questions, will be analysed using the conventional content analysis (CCA) to establish the main underlying factors contributing to implementation of specific policies.

All indicators will be tested for validity and reliability and most importantly for the ease with which they can be generated using routine and administrative data. A dashboard or score card will be developed to display performance or progress of the first wave barometer assessment. Simple info-graphics will be developed in ways that allow for easy updates and analysis of future trends.

The value of a well-functioning PIB is that it may extend the value chain and transcend the current health issues, thus adding ‘forecasting’ to the (A) Prevention, (B) Treatment, and (C) Follow up chain. This includes identification of and planning for future health issues and using the barometer to measure the country’s effectiveness in terms of addressing them.

### Data dissemination and stakeholder engagement

Unlike traditional studies in which results are simply published in the form of technical reports and journal articles, the results of the first wave barometer survey will be used to actively engage with upstream, midstream and downstream stakeholders to effect necessary changes.

### Study limitations

The study is multi-faceted and is scheduled to be implemented in a few selected districts and not at all the districts. It might be possible that relevant contextual factors and actors might inadvertently be missed. However, the study will use secondary information from many more districts to assess implementation. The selected policies are not mutually exclusive meaning that some issues may overlap. However, attempts will be made to ensure that overlapping issues are isolated and discussed.

## Ethical considerations

All respondents to the study will sign consent forms that clearly indicate how confidentiality will be protected in the study. No names will be recorded and all participants will be coded (anonymised) before analysis and reporting of results. No one will be forced to participate and refusals will be recorded as such. The phone contacts of the participants will not be shared with others outside of this study and will only be used for delivery of the survey. Phone contacts and all identifiers (such as titles, office names, etc.) will be will be stripped off the data to avoid tracing responses to a particular interviewee. Although a token of appreciation €10.0 will be provided to on-line interviewees, this is to compensate for the cost of data bundles that will be used by the respondents while responding to the survey. This token payment will not be used to coerce the respondents – although it will be offered at the point when the respondent terminates the interview. The phone number to receive this token will be provided at the point of exit from the online survey. These phone numbers will be used only for paying the token and not linked to the data a respondent has provided during the survey.

## Discussion

We anticipate that this study will have two key contributions to the body of knowledge on policy implementation in Uganda and other low and middle income countries. First, it is the first known attempt to develop a systematic methodology for assessing progress in policy implementation and at the same time engaging relevant stakeholders for corrective action. So not only is the study seeking to identify gaps and constraints but to also facilitate effective policy implementation for impact. The idea is to have three waves of such barometer surveys during the life of the SPEED project which will allow for horning of the protocol in terms of stakeholder engagements, measurement metrics (both qualitative and qualitative), sources of data both primary and secondary and also use of online tools for wider involvement of participants and also for quick processing of the data for further engagements. Second, the study results will contribute to achievement of UHC objectives for Uganda by ensuring that the selected policy cases for the barometer are relevant to the UHC agenda and government priorities. Application of an accountability framework such as a policy implementation barometer will foster a sense of responsibility amongst implementers and upstream monitoring will therefore become more effective and hopefully the impact will improve.

Policies succeed or fail for a variety of reasons and this study seeks to unpack the drivers and bottlenecks in the implementation of specific policies towards UHC in Uganda−that is malaria, family planning and emergency obstetric care. These are transnational priority health areas affecting most countries in the sub-region meaning that the results of this study will potentially have far reaching benefits. We anticipate that the overall structural and systemic challenges of policy implementation will influence regional policy implementation support networks and agencies. A toolkit on the policy implementation barometer and a validated database of appropriate measurement metrics will be developed as by-products of the study. Clearly, there is no silver bullet to addressing policy implementation as countries differ in contexts and contexts change over time. What is critical is to have a system or culture of learning from implementation which the PIB seeks to engender in Uganda.

## Abbreviations

ABC: Abstinence, Behaviour Change, and Condoms
ANC: Antenatal care
ART: Anti-retroviral therapy
CCA: Conventional Content Analysis
DHIS: District health information system
DHMT: District Health Management Teams
DHO: District Health Office
EPI: Expanded Programme on Immunisation
HIV: Human Immunodeficiency Virus
ICCM: Integrated Community Case Management
IRS: Insecticide Residual Spraying
ITN: Insecticide-treated nets
MakSPH: Makerere School of Public Health
MOH: Ministry of Health
PIB: Policy Implementation Barometer
PIBA: Policy Implementation Barrier Assessment
PMTCT: Prevention of Mother to Child Transmission
SPEED: Supporting Policy Engagement for Evidence-based Decisions
TOC: Theory of change
UHC: Universal Health Coverage
